# Medical Society of the County of Erie

**Published:** 1897-04

**Authors:** 


### Society Proceedings.

### MEDICAL SOCIETY OF THE COUNTY OF ERIE.

Reported by FRANKLIN C. GRAM, M. D., Secretary.

### IN MEMORY OF DR. ALPHONSE DAGENAIS.

A SPECIAL meeting of the Medical Society of the County of Erie was held in the rooms of the Buffalo Academy of Medicine, 619 Main street, on the evening of March 5, 1897.

In the absence of the president and vice-president the secretary called the meeting to order, and Dr. Lucien Howe was elected temporary chairman.

Dr. Howe stated that the society had met to pay tribute to the memory of Dr. Alphonse Dagenais, who died March 4, 1897. He considered it eminently proper to meet and show respect to every member who has departed. In the rush of life a man falls in the ranks, the ranks close up and move on as if nothing had happened. Let us pause a moment to pay respect to one who was an examplar of professional worth and good citizenship.

The following letters were read by the secretary :

*Dear Doctor Gram:*

I regret that it will be impossible for me to attend the meeting of the Medical Society of the County of Erie this evening, and that I am thus deprived of the sad privilege of paying tribute to the memory of our deceased friend, Dr. Dagenais.

He was a man of uncommon worth, of great professional and moral rectitude, a conscientious physician and a useful citizen.

The profession and the community are great losers in his taking off, and a loving family is deeply, grievously afflicted. Let us mingle our tears with theirs and offer the feeble comfort of sympathy in this greatest of human trials. Very sincerely yours,

WILLIAM WARREN POTTER.

284 Franklin street, Thursday, March 5, 1897.

BUFFALO, N. Y., February 5, 1897.

*Dr. F. C. Gram, Secretary :*

DEAR SIR—I regret that a previous engagement prevents me from being present at the meeting this evening to take action on the death of Dr. Dagenais.

I cannot, however, let the occasion pass without paying tribute to the high character of Dr. Dagenais as a physician, a man and a citizen. Whenever I have met him, professionally or socially, I have always found him in all respects such a one as we like to know. The profession has been deprived of a valuable member, the city of a useful citizen and his friends of one dear to them.

Yours very truly,

DELANCEY ROCHESTER.

BUFFALO, March 5, 1897.

*Chairman Special Meeting of the Erie County Medical Society :*

DEAR DOCTOR—I desire to express regret at my inability to be present tonight. More particularly during the last few years I have learned to know Dr. Dagenais and to recognise his good qualities. His kindness of heart, as shown in his intercourse with his patients, will cause very many to mourn his death as a severe personal loss.

Sincerely yours, P. W. VAN PEYMA.

On motion the chair appointed Drs. Ingraham, Rochester and Ernest Wende, to draft a memorial. The committee submitted the following :

MEMORIAL OF DR. ALPHONSE DAGENAIS.

For twenty-seven years Dr. A. Dagenais has been engaged in the active practice of medicine in Buffalo, and during this long period not an unkind word has ever been spoken concerning him by his colleagues or his clients. On the contrary, he acquired a large practice by careful attention to the welfare of his patients, to whom he was especially endeared.

His methods were strictly ethical, his manners courteous and his practice professional in the highest sense.

Called hence in the midst of his ripest years and best work, he is sadly missed by the profession, by his patients whose numbers are large, by the community in which his citizenship was unstained, and, lastly, by a loving wife and daughter, whose bereavement is the keenest and whose grief we dare not disturb, except with words of kindest sympathy.

*Resolved*, That the foregoing memorial be communicated to the mourning family, to the BUFFALO MEDICAL JOURNAL, and to the newspaper press.

(Signed) H. D. INGRAHAM,
DE LANCEY ROCHESTER,
ERNEST WENDE.

Remarks were made by Drs. Ingraham, Dunham, Eugene Smith, Gram, Clothier and the chairman, after which the meeting adjourned.

---

### IN MEMORY OF DR. JAMES B. SAMO.

The Medical Society of the County of Erie met in special session Sunday, March 14, 1897, at 4.30 o'clock P. M.

Dr. JOHN HAUENSTEIN, now the oldest surviving member of the profession in Buffalo, was elected chairman, and on assuming the duties stated that the society had been called together to pay a final tribute to the memory of Dr. James B. Samo, who died March 13, 1897, at the ripe age of 85 years. He recalled some of his experiences with the deceased, which extended over a period of many years.

A committee, consisting of Drs. Lucien Howe, Thomas Lothrop and J. B. Coakley, was appointed to submit a memorial, which reported as follows :

### MEMORIAL OF DR. JAMES B. SAMO.

It is fitting that as members of the Medical Society of the County of Erie some record should be made of the death of one of the oldest practitioners among us. Dr. James B. Samo came here when the city was almost in its infancy, and has taken a more or less active part in the professional growth of a typical American town, from a village to one of the largest cities of the country. He has served faithfully during two cholera epidemics, facing contagion in its worst forms, and in a quiet but efficient manner combating a disease that made more havoc with human life than any other form of epidemic which we ever encounter now. This alone would entitle him to be enrolled among the veterans of medical practice, and as such we honor his service and pay this mark of respect to his memory. In addition we feel that his example has not been without its influence upon those with whom he came in contact. His gentleness of nature was carried to an extreme which bordered on diffidence, but his kindly heart was always open to the appeals of those whom he could serve, and he never failed to give such aid as he could without thought of pecuniary reward.

We therefore unite in paying this tribute of respect to a practitioner of long and faithful service, a man of kindly impulses and one generous to a fault.

Signed, LUCIEN HOWE, THOMAS LOTHROP,
J. B. COAKLEY.

The memorial was adopted and it was decided to attend the funeral in a body.

Remarks were made by some of the members present. Dr. W. W. Potter said that while he did not enjoy an intimate acquaintance with Dr. Samo, yet he had known him for more than thirty years and had a kindly feeling for his memory. Few, perhaps, knew him intimately, as he was one of those quiet, unassuming men, who never seek prominence. Yet the record he made was most remarkable. A vice-president, then president of this society, and subsequently its librarian for a period of forty years! He must have been more than an ordinary man to retain the confidence of his medical brethren for more than a generation!

Dr. Thomas Lothrop gave a brief history of Dr. Samo's last illness. At the ante-mortem request of the deceased an autopsy was held, which revealed a complete occlusion of the common bile duct and a primary cancer of the neck of the gall bladder. Although Dr. Samo had not been a success financially, because he did not have the confidence in himself which his knowledge deserved, yet he fell into the hands of the Good Samaritan and his last days were rendered very comfortable at the Church Home, where the greatest possible care was bestowed upon him. Dr. Lothrop also spoke of his good character and his worth as a man.

Dr. Coakley had known him for years and spoke of his calm dignity and gentlemanly bearing under all circumstances.

Dr. Howe stated that Dr. Samo had inquired, when on his deathbed, whether the doctors would hold a little meeting in his memory after he was gone, and when assured that such would be done the old gentleman seemed content. We cannot judge of him, said Dr. Howe, any more than youth can judge of age. He came on the stage years ago, and was the last of the noted physicians of that period. His name was associated with those of Hamilton, Flint, White and others, honored and renowned, and long since passed away. We of the present, with our knowledge of disease germs, have little conception what it was to go through cholera epidemics, such as invaded this city in 1853–4. We have words of praise for the soldier who stares death in the face, but never a word about that soldier who moves noiselessly about constantly fighting death. Dr. Samo was one of those whose steadfast courage was manifested during the epidemics mentioned.

After a few words from Dr. Dunham, the society determined by vote to attend the funeral in a body and then adjourned.

---

### IN MEMORY OF DR. FREDERIC W. BARTLETT.

The Medical Society of the County of Erie assembled in special session, March 18, 1897, at 8 P. M., in the rooms of the Buffalo Academy of Medicine, to take appropriate action on the death of Dr. Frederic W. Bartlett.

Dr. JOHN CRONYN was chosen to preside, and announced the death of Dr. Frederic W. Bartlett, which had occurred March 17, 1897.

On motion a committee, consisting of Drs. Hubbell, Wetmore and Barnes, was appointed to draft appropriate resolutions. The committee submitted the following, which was unanimously adopted:

#### MEMORIAL OF DR. F. W. BARTLETT.

In the death of Dr. Frederic W. Bartlett this society and the medical profession have lost an earnest, active and able member. He was a constant attendant upon medical meetings, was an efficient officer and was original and progressive in his views on medical subjects. He was not only pervaded by the spirit which should actuate the true physician as relates to the medical profession, but was also unselfishly devoted to the welfare of his patients at whatever personal sacrifice. He was everywhere known as a genial, public-spirited and conscientious member of the community.

*Resolved*, That this memorial be incorporated in the minutes of this society and that a copy be transmitted to the family of the deceased.

*Resolved*, That this society attend the funeral in a body.

A. A. HUBBELL,
S. W. WETMORE,
E. R. BARNES,
*Committee.*

Speaking on the adoption of the memorial, Dr. Benedict, as one of the younger members of the profession, laid stress on the cordiality with which Dr. Bartlett treated the younger physicians. He did not believe that Dr. Bartlett had ever refused a patient on the ground of poverty, but had on the ground of dishonesty. His wonderful power of endurance often put to shame many a younger man.

Dr. Lucien Howe said that we should ever show our sympathy by our presence on such occasions as these. We are "the stuff that dreams are made of, and our little lives are rounded with sleep."

Dr. Thomas Lothrop was present to show his appreciation for the departed member. He had known Dr. Bartlett as a tireless worker, harder, perhaps, than any other man in Buffalo, who had, probably kept up his work too late in life—a warning to many others. He had used up his vital powers, so that there was no resistance left when the final attack came. He was large-hearted, and did much through motives of charity. Yet there should be a limit. We expect the younger members to do much charitable practice, but the older ones should graduate from it for two reasons : to give the younger ones a chance, and because we cannot afford that kind of work. He was a good general practitioner, well informed, and had good success. His death was a loss to the community and also to the profession. Dr. Lothrop deprecated the lack of interest generally taken in meetings of this kind.

Dr. Cronyn stated that he knew Dr. Bartlett longer than anyone else in this city, that both came to Buffalo about the same time, fifty-three years ago, and for a long period were close neighbors, and always remained fast friends. He related some professional and social incidents out of their respective careers. No one who ever placed himself under Dr. Bartlett's care died for want of attention. He would often go to see patients three or four times daily whom most others would have thought quite sufficient to visit every other day.

Dr. Hubbell said the deceased was a leader in his profession. No man was so regular at medical meetings, or took a greater interest in the proceedings. He had many distinctive ideas, attractive characteristics, and certain qualities of geniality. He knew him intimately for the past few years, and knew the interest he took in the community and the profession. Dr. Bartlett was one of the first, if not the first, to propose the idea that Buffalo should have an Academy of Medicine, which finally became a successful fact. He gave most liberally towards this, and believed that the Academy should have a permanent home of its own. Toward this end he had worked out a feasible plan. His death is a great loss to the medical profession.

Dr. Callanan also spoke of the great consideration which Dr. Bartlett showed his poorer patients, and recalled some pleasant experiences.

Dr. GOLTRA desired to pay a tribute of respect to one whom he had met more frequently than any one else at the bedside. Two prominent qualities impressed him: as a natural born gentleman; and his untiring attention to his patients.

Dr. GRAM spoke of his pleasant associations with the deceased as the presiding officer of this society.

Dr. WETMORE referred to the deceased as a Christian gentleman of the old school. Unwavering in his religious belief, conscientious, kind and obliging, social in his greeting, true to his friends, his neighbors, his profession, himself, his family and his God.

The society then adjourned.

LEGISLATION AT ALBANY.

A SPECIAL MEETING of the Medical Society of the County of Erie was held at the Buffalo Academy of Medicine, March 16th, at 8 P. M., to consider legislation at Albany.

The meeting was called to order promptly at the time named in the card, as a regular meeting of the Academy was to be held at the same place at 8.30 o'clock.

Among the members present were Drs. Lucien Howe, H. R. Hopkins, Edward Clark, Jacob Goldberg, W. W. Potter, W. G. Bissell, Benedict, Wyckoff, Renner, C. S. Jewett, Gram, Pohlman, Dowd, Hartwig, Waterman, Finerty, Grant, Satterlee, Clothier, Milliner and others.

Dr. HENRY R. HOPKINS was elected chairman, and the secretary read the following request for a call of the meeting:

*To the Secretary of the Medical Society of the County of Erie:*

SIR—The undersigned members of said society respectfully request that a special meeting be called for Tuesday evening, March 16, 1897, at 8 o'clock, for the purpose of considering legislation introduced into the legislature at Albany relating to state medical examinations.

Yours truly,

ERNEST WENDE. EDWARD CLARK.
WM. W. POTTER. JOHN PARMENTER.
HENRY R. HOPKINS. JACOB GOLDBERG.
LUCIEN HOWE.

BUFFALO, March 12, 1897.

Dr. W. W. POTTER read a copy of a bill introduced into the legislature which proposes to amend Section 141 of the state medical practice act so that it shall read as follows:

SECTION 141. *State Board of Medical Examiners.*—There shall continue to be three separate state boards of medical examiners of seven members each, each of whom shall hold office for three years from August first of the year in which appointed. One board shall represent the Medical Society of the State of New York and the New York State Medical Association, one the Homeopathic Medical Society of the State of New York and one the Eclectic Medical Society of the State of New York. Each of these four [three] societies shall at each annual meeting nominate twice the number of examiners to be appointed in that year on the board representing it. The board representing the Medical Society of the State of New York and the New York State Medical Association shall be composed of members from each of such organisations, to be appointed as follows: At the annual session of the New York State Medical Association held during the year eighteen hundred and ninety-seven, nominations shall be made for all the vacancies occurring in such board, by expiration of term, during the year eighteen hundred and ninety-eight; and thereafter the New York State Medical Association and the Medical Society of the State of New York shall each be entitled annually to the appointment of one member. In the years when three members of such board are to be appointed, the appointment of the extra member shall alternate between such two organisations; but when three members of such board are next to be appointed, the extra member shall be appointed from nominations made by the New York State Medical Association. All vacancies occurring otherwise than by expiration of term shall be filled by appointment from the organisation to which the member belonged whose office is vacated. The names of [such] nominees for appointment to such state boards shall be annually transmitted under seal by the president and secretary prior to May first to the regents, who shall, prior to August first, appoint from such lists the examiners required to fill any vacancies that will occur from expiration of term on August first. Any other vacancy, however, occurring shall likewise be filled by the regents for the unexpired term. Each nominee, before appointment, shall furnish to the regents proof that he has received the degree of doctor of medicine from some registered medical school, and that he has legally practised medicine in this state for at least five years. If no nominees are legally before them from a society the regents may appoint from members in good standing of such society without restriction. The regents may remove any examiner for misconduct, incapacity or neglect of duty.

SEC. 2. This act shall take effect immediately.

The following preamble and resolutions were then adopted:

WHEREAS, The Medical Society of the State of New York, since 1806, has been the representative body of the medical profession of the state, constituted as such by statutory law that has been amended from time to time to meet the changing conditions that have arisen during the present century ; and

WHEREAS, The state society, aforesaid, is made up of delegates and permanent members sent from the several county medical societies, which county societies are therefore an integral part of the State society ; and

WHEREAS, All legal practisers of medicine in the State of New York must be members of the several county medical societies thereof, hence exercise a voice each for himself in the proceedings and action of the state medical society aforesaid ; and

WHEREAS, The medical profession of the State of New York, acting through the county medical societies and the state medical society aforesaid, at great personal inconvenience and expense, after eight years of constant effort, succeeded in persuading the Legislature to enact a law which is administered by the Regents of the University, establishing a state examination for license to practise medicine in this state ; and

WHEREAS, This state examination and license fixes a qualification for the medical profession of the future in this state ; and

WHEREAS, The interests of the public health, and the competency of the medical profession, are indissolubly united ; and

WHEREAS, Senate Bill, No. 671 (or Assembly Bill, No. 1255), which we have heard read in our presence, proposes a radical change in the manner of the appointment of the members of one of the three separate state boards of medical examiners ; therefore, be it

*Resolved*, That the Medical Society of the County of Erie is unalterably opposed to the legislation proposed by the bill or bills aforesaid ; and further, be it

*Resolved*, That we call upon the members of the Senate and Assembly representing the county of Erie in the present legislature, to use all honorable means in opposing its passage, and in preventing it from becoming a law ; and further, be it

*Resolved*, That a committee consisting of Drs. Edward Clark, Ernest Wende and William Warren Potter, all of Buffalo, be and is hereby appointed to attend a hearing or hearings at Albany, that may be granted by the senate or assembly committees having such bill or bills in charge, said committee to have full power to act for the medical society of the county of Erie in presenting its opposition to these bills to the legislature.

The meeting then adjourned.

HENRY R. HOPKINS, M. D.,
*Chairman, pro tem.*

FRANKLIN C. GRAM, M. D., *Secretary.*

